# Comprehensive investigation into cuproptosis in the characterization of clinical features, molecular characteristics, and immune situations of clear cell renal cell carcinoma

**DOI:** 10.3389/fimmu.2022.948042

**Published:** 2022-10-06

**Authors:** Bao Wang, Qiang Song, Yuang Wei, Xiangzheng Wu, Tian Han, Hengtao Bu, Sensheng Tang, Jian Qian, Pengfei Shao

**Affiliations:** Department of Urology, The First Affiliated hospital of Nanjing Medical University, Nanjing, China

**Keywords:** copper-induced cell death, cuproptosis, ccRCC, prognosis, tumor microenvironment, immunotherapy

## Abstract

**Background:**

Copper-induced cell death has been widely investigated in human diseases as a form of programmed cell death (PCD). The newly recognized mechanism underlying copper-induced cell death provided us creative insights into the copper-related toxicity in cells, and this form of PCD was termed cuproptosis.

**Methods:**

Through consensus clustering analysis, ccRCC patients from TCGA database were classified into different subgroups with distinct cuproptosis-based molecular patterns. Analyses of clinical significance, long-term survival, and immune features were performed on subgroups accordingly. The cuproptosis-based risk signature and nomogram were constructed and validated relying on the ccRCC cohort as well. The cuproptosis scoring system was generated to better characterize ccRCC patients. Finally, *in vitro* validation was conducted using ccRCC clinical samples and cell lines.

**Result:**

Patients from different subgroups displayed diverse clinicopathological features, survival outcomes, tumor microenvironment (TME) characteristics, immune-related score, and therapeutic responses. The prognostic model and cuproptosis score were well validated and proved to efficiently distinguish the high risk/score and low risk/score patients, which revealed the great predictive value. The cuproptosis score also tended out to be intimately associated with the prognosis and immune features of ccRCC patients. Additionally, the hub cuproptosis-associated gene (CAG) FDX1 presented a dysregulated expression pattern in human ccRCC samples, and it was confirmed to effectively promote the killing effects of copper ionophore elesclomol as a direct target. *In vitro* functional assays revealed the prominent anti-cancer role of FDX1 in ccRCC.

**Conclusion:**

Cuproptosis played an indispensable role in the regulation of TME features, tumor progression, and long-term prognosis of ccRCC.

## Introduction

The term “apoptosis” was first raised up in 1972 by John et al. to describe a form of programmed cell death (PCD) with morphologically observed changes and physiological dysregulation of cells (1). The emergence of apoptosis triggered a series of studies on regulated cell death (RCD) which is continuous to today. We have known that RCD, a universal process in living organisms that is essential for tissue homeostasis or restoring biological equilibrium following stress, is distinct from the conventional pathological cell death like necrosis. Over years, it has been successively reported in the non-apoptotic form of lysosomal cell death (2000), pyroptosis (2001), necroptosis (2005), immunogenic cell death (2005), entosis (2007), ferroptosis (2012), autosis (2013), etc. (2). Increasing studies have provided us fundamental insights into understanding the mechanisms underlying these RCDs, and we come to establish the connections between certain cell death and human diseases, including autoimmune diseases, infections, inflammatory diseases, and cancers (3, 4). It is also well learned that various RCDs initiate themselves with different substrates, intermediates, effectors, working systems, and molecular events (5).

Recently, Tsvetkov and colleagues have reported a novel form of RCD mediated by the dysregulated accumulation of *in vivo* copper (Cu). In the study, the authors noticed a situation where even a modest intracellular concentration of Cu can be toxic and result in cell death. They ultimately discovered an ancient mechanism that initiated the copper-induced cell death, protein lipoylation. The copper-induced cell death was recognized to be distinct from all other known mechanisms of RCD (e.g., apoptosis, ferroptosis, necroptosis), and this kind of uncharacterized cell death mechanism was termed “cuproptosis” (6).

Here, tremendously interested in the cuproptosis study, we decided to explore the significance of copper-induced cell death in the typical genitourinary tumor, clear cell renal cell carcinoma (ccRCC). ccRCC is the most common type of kidney cancers and accounts for more than 80% of renal cell carcinomas with an increased incidence worldwide. Yet, neither the pathogenesis of ccRCC, nor the prognostic biomarkers applicable for clinical usage, has been fully investigated. Given the active vulnerability of this disease to ferroptosis (7), apoptosis (8), necroptosis (9), and other RCD forms, we proposed that the copper ion and derived-cell death may play an indispensable role in ccRCC.

In the present study, we made a comprehensive online study on cuproptosis-related genes to explore their relationship with ccRCC. Through multiple uses of gene clustering, survival analysis, enrichment analysis, and immune-related investigation, we successfully recognized the prognostic significance of cuproptosis and established the cuproptosis score for estimating the long-term survival and treating the response of ccRCC patients. We believe that this study holds great potential to bring novel insights into understanding the copper-induced cell death in human cancer, especially in ccRCC.

## Materials and methods

### Data sourcing and preprocessing

Normalized data of gene expression (in the format of FPKM), somatic mutation, CNV files, and corresponding clinicopathological information of ccRCC patients were collected from The Cancer Genome Atlas (TCGA, https://portal.gdc.cancer.gov). Ten cuproptosis-associated genes (CAGs) were derived from the study of Tsvetkov et al. (6). Clinical information of patients included age, sex, TNM stage, stage, grade, follow-up time, and survival status. The FPKM values were normalized to transcripts per kilobase million (TPM) for further analysis.

### Consensus clustering analysis

R package “ConsensusClusterPlus” was applied for consensus unsupervised clustering analysis, and ccRCC patients from TCGA-KIRC database were classified into distinct molecular subgroups based on CAG and differentially expressed gene (DEG) expression patterns. The *k*-means algorithm was used to determine the optimal grouping number.

### Survival analysis and receiver ROC curves

The Kaplan–Meier (K–M) method was used to assess the overall survival (OS) between different groups based on gene expression, risk signature or cuproptosis score. The receiver operating characteristic (ROC) curves were depicted using the “survival-ROC” package in R, and the areas under the curve (AUC) were calculated to judge the specificity and sensitivity of survival analysis.

### Identification of differentially expressed genes

The “limma” package in R was used to identify DEGs between three subgroups derived from CAG clustering. Genes with an adjusted p-value < 0.05 and |log2(FC)| > 1.0 were considered to be significantly differentially expressed. Finally, a total of 1,117 genes were characterized and subjected to further analysis.

### Gene set enrichment analysis

Gene set enrichment analysis (GSEA) was performed to analyze the functional pathways and biological processes variation between groups. Gene sets associated with various hallmarks were downloaded from the Molecular Signatures Database (MSigDB, http://software.broadinstitute.org/gsea/msigdb/). Gene Ontology (GO) and Kyoto Encyclopedia of Genes and Genomes (KEGG) analyses were conducted accordingly to identify functional enrichment differences between groups. Sets of gene signatures of immune cell infiltration, immune functions, immunotherapy, and therapeutic response markers were subjected to the single-sample GSEA (ssGSEA) analysis. The enrichment score of each gene signature was calculated through gene set variation analysis (GSVA) using the “GSVA” R package. Only terms with a *p*-value < 0.05 were selected and shown.

### Immune cell infiltration and immune score analysis

The CIBERSORT algorithm was used to determine the proportion of immune cell subsets in each TCGA-KIRC sample. The ESTIMATE method was used to generate stromal and immune scores to evaluate the tumor purity and distribution of cell types in tumor environment (TME).

### Construction of the risk signature and nomogram

Univariate Cox regression analysis was conducted on DEGs achieved above with a calculated HR. The Least Absolute Shrinkage and Selection Operator (LASSO) Cox regression analysis was used to calculate the weighting coefficient and construct the risk model using the “glmnet” package in R. After being divided into high- and low-risk groups upon the optimal cutoff of risk score, patients were analyzed for the survival rate using the Kaplan–Meier method. At last, the multivariate Cox regression analysis was utilized to integrate the gene signature and clinical factors into the nomogram.

### Development of the cuproptosis score

To quantify the cuproptosis pattern of each ccRCC patient, the cuproptosis score was calculated. The Boruta algorithm was utilized to reduce the size of cuproptosis gene signatures A and B, and principal component 1 was extracted by principal component analysis as a signature score. Each patient’s score was then calculated: cuproptosis score =∑PC1A-∑PC1B, where PC1A represents the first component of feature A and PC1B represents the first component of feature B. The cuproptosis score of the molecular patterns or gene clusters was assessed by the Kruskal–Wallis test.

### Immunotherapy response and drug sensitivity estimation

The Genomics of Drug Sensitivity in Cancer (GDSC; https://www.cancerrxgene.org/) database was applied to predict patient response to several agents. The IC50 value was quantified using the “pRRophetic” package in R. The response to immune checkpoint blockade (ICB) was estimated through the online tool Tumor Immune Dysfunction and Exclusion (TIDE, http://tide.dfci.harvard.edu/login/) method, and the TIDE scores were compared between subgroups. The immunophenotype score (IPS) was calculated to detect the immune-related treatment response in the compared subgroups.

### Human tissue sample and real-time polymerase chain reaction

A total of 22 paired human ccRCC clinical samples of tumors and adjacent tissues were obtained through radical nephrectomy on patients. Each patient was clinically diagnosed as ccRCC by at least two independent pathologists. All patients were properly informed, and the consents were acquired accordingly. The study was approved by the ethics committee of our hospital.

To detect the FDX1 mRNA level in human clinical samples, total RNA was extracted from ccRCC samples by TRIzol Reagent (Thermo Fisher Scientific, Waltham, MA, United States) and reversely transcribed into cDNA following the manufacturer’s protocol. Then, the quantitative real-time polymerase chain reaction (qRT-PCR) was conducted on a LightCycler 480 II (Roche Diagnostics, Basel, Switzerland) instrument using the SYBR Green Master Kit (Vazyme, Nanjing, China). The primers used to amplify CAGs were purchased from Tsingke Biotechnology (Beijing, China). The sequences of primers were as follows: FDX1: 5′-GAGGGAACCCTGGCTTGTTC-3′ (forward) and 5′-CAGGAGGTCTTGCCCACATC-3′ (reverse); *β*-actin: 5′-CCCATCTATGAGGGTTACGC-3′ (forward) and 5′-TTTAATGTCACGCACGATTTC-3′ (reverse). Each qRT-PCR was performed in triplicate, and β-actin was used to normalize gene expression.

### Cell culture and plasmid vector transfection

Human ccRCC cell lines 786-O and 769-P were obtained from the Cell Bank of the Chinese Academy of Sciences (Shanghai, China). The cells were cultured in 1640 medium with 10% fetal bovine serum (FBS) in an environment of 5% CO_2_ and 37°C. A plasmid vector carrying FDX1 sequences to fulfill gene overexpression was transfected into cells using Lipofectamine 3000 Reagent (Invitrogen, United States). The plasmid was purchased from GeneChem (Shanghai, China), and detailed information was provided in the **Supplementary Material**. qPCR was used to evaluate the overexpressing efficiency. After that, the stably FDX1-overexpressed cell lines 786-O-OE and 769-P-OE were established and applied for further experiments.

### Western blotting

Total proteins were extracted from cells transfected with plasmids using radioimmunoprecipitation assay (RIPA) buffer containing protease inhibitors. BCA Protein Assay Kit (Beyotime Biotechnology, Shanghai, China) was used to determine the protein concentration. Proteins were separated by 10% sodium dodecyl sulfate–polyacrylamide gel electrophoresis (SDS-PAGE) and transferred to a polyvinylidene difluoride (PVDF) membrane. After being blocked within skim milk for 3 h, the membranes were incubated overnight with the primary antibody specifically against FDX1 at 4°C. After that, membranes were incubated with the secondary antibody anti-mouse IgG (Cell Signaling Technology, Danvers, MA, United States) for 2 h. Tubulin expression was used as a loading control. Protein bands were visualized with an enhanced chemiluminescence (ECL) detection system (Thermo Fisher Scientific, Rochester, NY, United States). Analysis was made using the ImageJ software (NIH, United States).

### Cell viability detection

Cell Counting Kit-8 (CCK8) assay was used to measure the viability or proliferative ability of ccRCC cells. The cells were resuspended, diluted, and seeded onto 96-well plates (1,500/well) and cultured in a 5% CO_2_ and 37°C environment. For cell viability, after cells were treated with elesclomol under a concentration of 20 nM (MCE, United States) for 12 h, 10 µl CCK8 Reagent (Solarbio, Japan) was added to each well and incubated for 1 h. For proliferative ability, at the time of 24, 48, 72, and 96 h, 10 µl CCK8 Reagent was added to each well and incubated for 1 h. After that, the absorbance at 450 nm for overexpression and control groups was measured accordingly.

### Cell migration capability detection

Transwell assay and wounding healing assay were performed to measure the migration capability of ccRCC cells. A total of 2*10^4^ cells were resuspended, diluted, and seeded into the upper chamber of a Transwell chamber (Corning Incorporated, United States) with 300 µl serum-free 1640 medium. The lower chamber was added with 700 µl 1640 medium containing 10% FBS. After being incubated in 5% CO_2_ and 37°C for 24 h, cells on the substrate of the membrane were fixed in 0.4% paraformaldehyde and stained with 0.1% crystal violet dye for 20 min at room temperature. Finally, after washing with PBS, cells were observed and photographed in randomly selected fields under a light microscope (Olympus Corporation, Japan).

### Statistical analysis

All statistical analyses were performed using R 4.1.0. Wilcoxon test was used for pairwise comparison. Kaplan–Meier analysis and log-rank test were used to compare the overall survival. More detailed statistical methods of transcriptome data processing are described in the above section. Statistical significance was set at two-sided *p* value <0.05.

## Results

### Recognition and genetic mutation of key cuproptosis-associated genes in ccRCC

**Figure 1:**
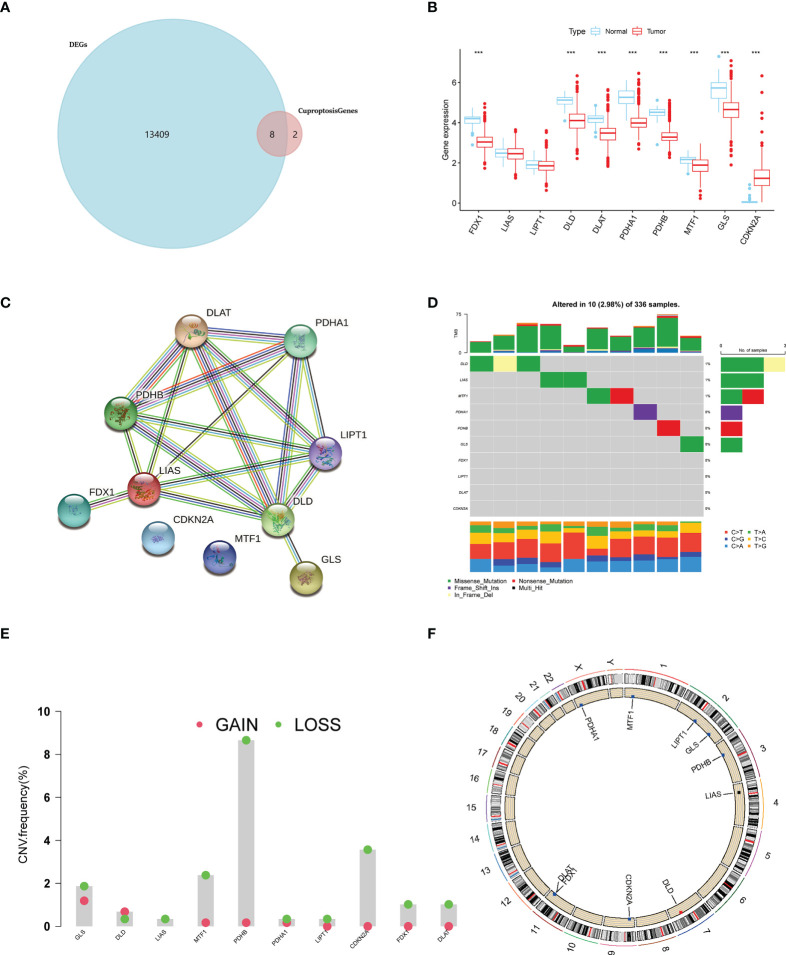
A landscape of key cuproptosis-associated genes (CAGs) in ccRCC. **(A)** Venn diagram of CAGs and ccRCC-derived DEGs. **(B)** Expression level of CAGs between tumors and normal tissues in ccRCC. **(C)** PPI network of CAGs. **(D)** Genetic mutation of CAGs in ccRCC samples. **(E)** CNA frequency of CAGs in ccRCC samples. **(F)** CNV sites of CAGs on chromosomes (*p* < 0.001 ***). DEGs: differentially expressed genes.

According to the study of Tsvetkov et al. (6), a total of 10 genes were identified as key genes most associated with copper-induced cell death, which were annotated as cuproptosis-associated genes (CAGs). To begin with, we sought for overlapping genes between CAGs and differentially expressed genes (DEGs) derived from paired or unpaired samples in TCGA-KIRC database. As a result, eight of the CAGs were recognized to be significantly differentially expressed in ccRCC **(** **Figure 1A** **)**. The expressing pattern revealed that seven of the CAGs were significantly more lowly expressed in tumors compared to normal kidney tissues (FDX1, DLD, DLAT, PDHA1, PDHB, MTF1, and GLS), while CDKN2A exhibited a significantly higher expression in tumors **(** **Figure 1B** **)**. The interactions between CAGs were then depicted using a PPI network **(** **Figure 1C** **)**. Next, we enquired the situation of genetic mutations of CAGs in 336 ccRCC samples, and the results showed a low incidence of genetic alteration (2.98%) **(** **Figure 1D** **)**. Moreover, the copy number variation (CNV) frequency was explored and PDHB obtained the highest frequency (8%) of CNV gain **(** **Figure 1E** **)**, and the mutation sites for CNV on the chromosome were labeled in the circle plot **(** **Figure 1F** **)**. These results preliminarily revealed the aberrant expression and significance of CAGs in ccRCC (*p* < 0.05 was considered statistically significant).

### Survival analysis on ccRCC patients based on CAGs

**Figure 2:**
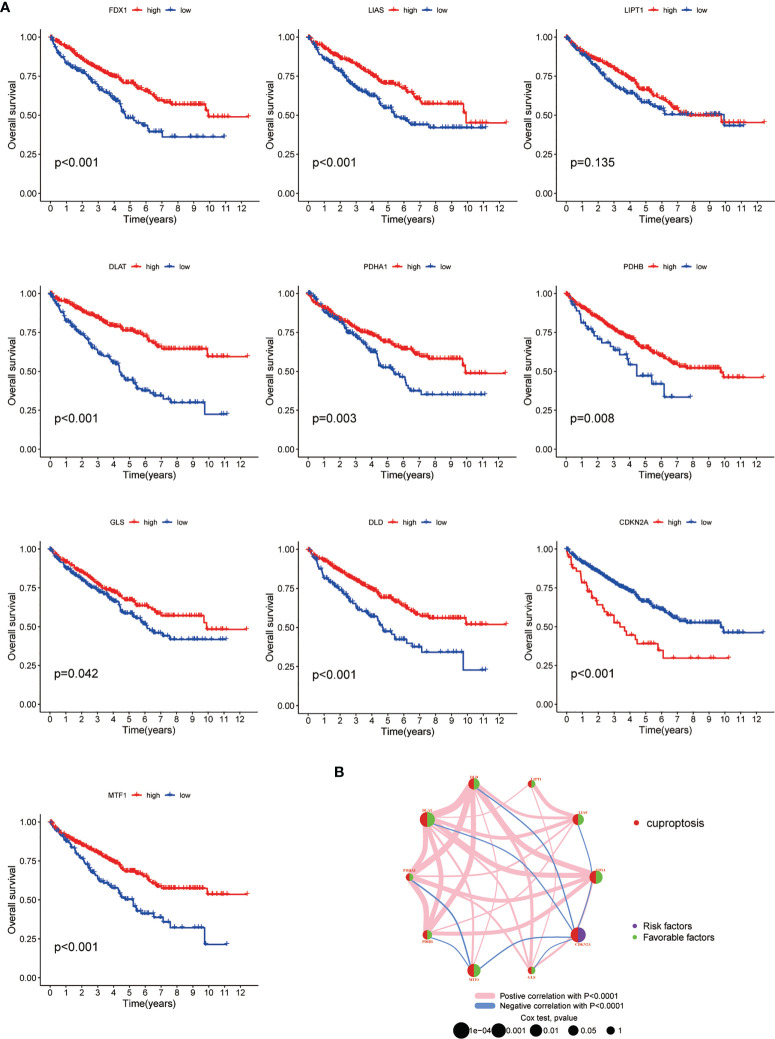
Survival correlation of CAGs in ccRCC patients. **(A)** Kaplan–Meier survival curves of 10 CAGs. **(B)** Interactive plot of 10 CAGs in the ccRCC cohort. The significance was labeled in different colors and sizes of dots (*p* < 0.05 *; *p* < 0.01 **; *p* < 0.001 ***. NS, not significant). CAGs: cuproptosis-associated genes.

Subsequently, to explore the prognosis relevance of CAGs in ccRCC, we made a survival analysis for each CAG on ccRCC patients from TCGA-KIRC database. Conformably, high levels of all CAGs, in addition to LIPT1 and CDKN2A, were significantly associated with poorer overall survival (OS) of ccRCC patients (*p* < 0.05) **(** **Figure 2A** **)**. When considering their expressing patterns in ccRCC, all CAGs serve as a favorable factor to the prognosis of ccRCC patients. Interestingly, contrary to other CAGs, CDKN2A had a higher expression in tumors while low-level CDKN2A was related to better OS, which made it a favorable factor to the prognosis of ccRCC patients as well. Besides, the significance of these regulators in the ccRCC cohort and interactions between them are depicted in **Figure 2B** (*p* < 0.05 was considered statistically significant).

### Generation and tumor immune microenvironment features of patient subgroups based on CAG clustering

**Figure 3:**
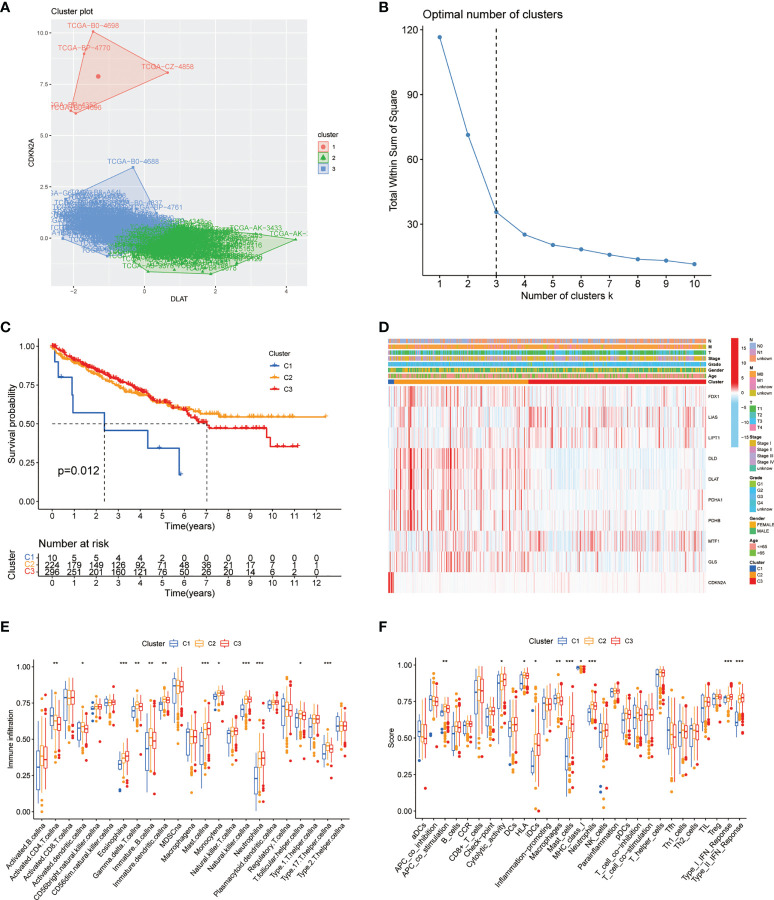
CAG pattern-based grouping of patients with distinct clinical and immune features. **(A)** PCA analysis indicates the separation of subgroups in the ccRCC cohort from TCGA database. **(B)** K-means curve suggests the optimal number of clusters. **(C)** Kaplan–Meier survival curves of three subgroups. **(D)** Heatmap of different clinicopathological features in three subgroups. Analysis of immune cell infiltration level **(E)** and immune-related function **(F)** between three subgroups. PCA: principal component analysis. TCGA: The Cancer Genome Atlas. KEGG: Kyoto Encyclopedia of Genes and Genomes. GSVA: gene set variation analysis (*p* < 0.05 *; *p* < 0.01 **; *p* < 0.001 ***. NS, not significant).

To better understand the potential role of CAGs in tumorigenesis and progression of ccRCC, the consensus clustering analysis was performed on the ccRCC cohort from TCGA-KIRC database. The optimal number of clusters turned out to be three, and a total of 530 patients were classified into three subgroups (designated as C1, C2, C3; N_C1_ = 10, N_C2_ = 224, N_C3_ = 296) based on their CAG expression patterns. The principal component analysis (PCA) confirmed the certain intergroup distribution **(** **Figures 3A, B** **)**. In the meantime, the following survival analysis validated the classification efficacy, with a significant gap of survival probability between C1 and C2/C3 subgroups (*p* < 0.05, **Figure 3C**). Next, significant differences of clinicopathological features were observed between each patient group, as shown in the heatmap, which demonstrated the distinct clinical background of patients divided by the CAGs **(** **Figure 3D** **)**. We also investigated the differences of immune cell infiltration level and vital immune processes between three subgroups. The results showed that 12 (represented by CD4^+^ T cells, T-helper cells, dendritic cells) of all 23 diverse immune cells displayed significantly different infiltration levels between subgroups **(** **Figure 3E** **)**, and several immune-related functions and processes (represented by mast cells, IFN response, regulation of antigen-presenting cells) displayed contrasting activity among three subgroups **(** **Figure 3F** **)**. Taken together, following the gene patterns of CAGs in ccRCC, patients were divided into three diverse subgroups, and relative analysis suggested that patients from different subgroups displayed distinct performance in clinical characteristics, immune cell infiltration and functions, and biological processes (*p* < 0.05 was considered statistically significant).

### Construction of the prognostic model based on DEGs from patient subgroups

**Figure 4:**
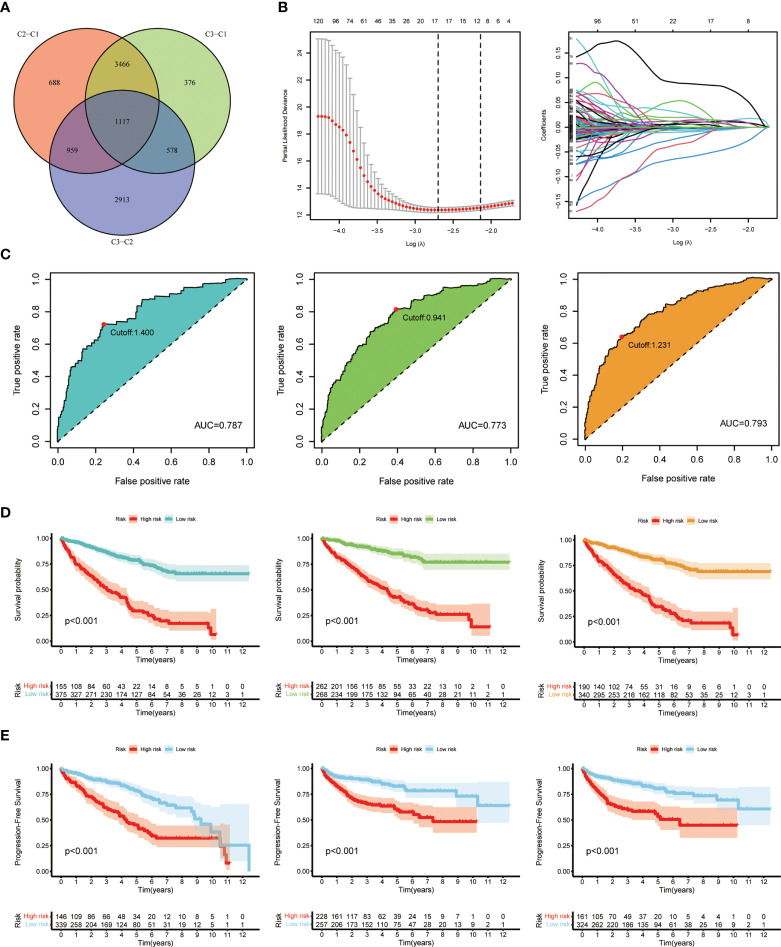
Generation and validation of the prognostic model. **(A)** Venn diagram of the DEGs *via* pairwise comparison among three subgroups. **(B)** LASSO regression analysis used to construct the prognostic model. **(C)** ROC curves for the predictive risk model of 1 year (left), 3 years (middle), and 5 years (right). **(D)** Overall survival analysis at the cutoff values: 1.400 (left), 0.941 (middle), and 1.231 (right). **(E)** Progression-free survival analysis at the cutoff values: 1.400 (left), 0.941 (middle), and 1.231 (right).

Considering the unignorable differences of biological phenotypes between three subgroups, we further made use of the intergroup differences to seek for prognostic benefits for ccRCC patients. First, a total of 1,117 DEGs were generated *via* pairwise comparison among three subgroups as previously described **(** **Figure 4A** **)**. Results of enrichment analysis including GO and KEGG are shown in the plots accordingly **(** **Figures S2A, B** **)**. These DEGs were then subjected to LASSO regression analysis to construct the risk signature, and the risk score for each ccRCC patient was calculated using the coefficients obtained from the LASSO algorithm **(** **Figure 4B** **)**. The formula of risk score was as follows: Risk Score = 0.1213 * FOXD2-AS1 + 0.0521 * SLC12A8 + (-0.0297) * CRB3 + 0.1289 * AC024060.2 + 0.0364 * XPOT + (-0.0884) * AR + (-0.0058) * CUBN + (-0.0523) * ITGA8 + (-0.1239) * KCNN3. Then, we analyzed the overall survival probability of 530 ccRCC patients from TCGA-KIRC database for 1, 3, and 5 years. Based on the cutoff values, patients were divided into low-risk and high-risk groups for 1-year (cutoff value = 1.400), 3-year (cutoff value = 0.941), and 5-year (cutoff value = 1.231) survival probability, and the ROC curves with AUC values for survival analysis are depicted in **Figure 4C**. In the meantime, according to the risk grouping of ccRCC patients based on cutoff values, we conducted analyses on overall survival (OS) (**Figure 4D**) and progression-free survival (PFS) (**Figure 4E**) of low- and high-risk patients, respectively. The results clearly exhibited the distinction of survival outcomes between low- and high-risk groups (*p* < 0.001). Taken together, these results suggested that the prognostic model based on cuproptosis-dividing genes had considerable prognostic efficacy and clinical value for ccRCC patients (*p* < 0.05 was considered statistically significant).

### Evaluation of the risk signature and clinical application

**Figure 5:**
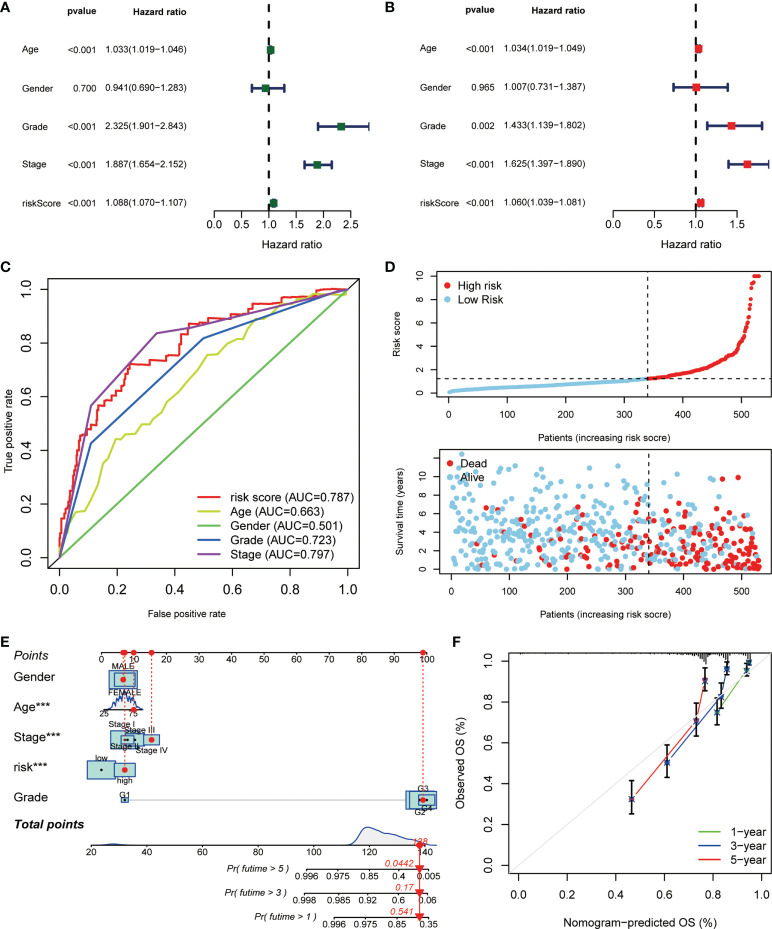
The correlation analysis of risk score and clinicopathological variables in ccRCC. The **(A)** uniCox and **(B)** multiCox analyses explored the independent prognostic value of risk score and multiple clinical factors. **(C)** The comparative ROC curves showed that the risk score was superior to most clinical factors in predicting patients’ long-term survival. **(D)** Ranked dot and scatter plots showing the risk score distribution and patient survival status. **(E)** Nomogram combining the risk signature and clinical factors. **(F)** Calibration curves for the nomogram-predicted OS at 1, 3, and 5 years (*p* < 0.001 ***).

Next, we made a comprehensive estimation on the risk signature achieved above. By including age, gender, grade, stage, and risk score, we conducted univariate (**Figure 5A**) and multivariate Cox (**Figure 5B**) regression analyses. As a result, the risk score turned out to be an independent protective factor for the prognosis of ccRCC patients, which was consistent with previous results. We also compared the predictive efficiencies of multiple variables as risk factors, and the results of ROC curves revealed the great predictive value of risk score in ccRCC (AUC = 0.787, **Figure 5C**). Considering the highest efficacy (AUC = 0.793) of the ROC curve at 5-year survival probability, we decided its cutoff value as the standard of patient grouping. The ranked dot map depicted the division of low- and high-risk groups among 530 ccRCC patients from the KIRC database, and the scatter plot showed the distribution of patients’ surviving status with different risk scores (**Figure 5D**). Finally, the nicely established prognostic model was depicted in a nomogram **(** **Figure 5E** **)**, and the calibration curve displayed certain concordance between the estimated survival probability and observed results for 1-, 3-, and 5-year OS **(** **Figure 5F** **)**.

### Generation and TIM characteristics of patient subgroups based on DEG clustering

**Figure 6:**
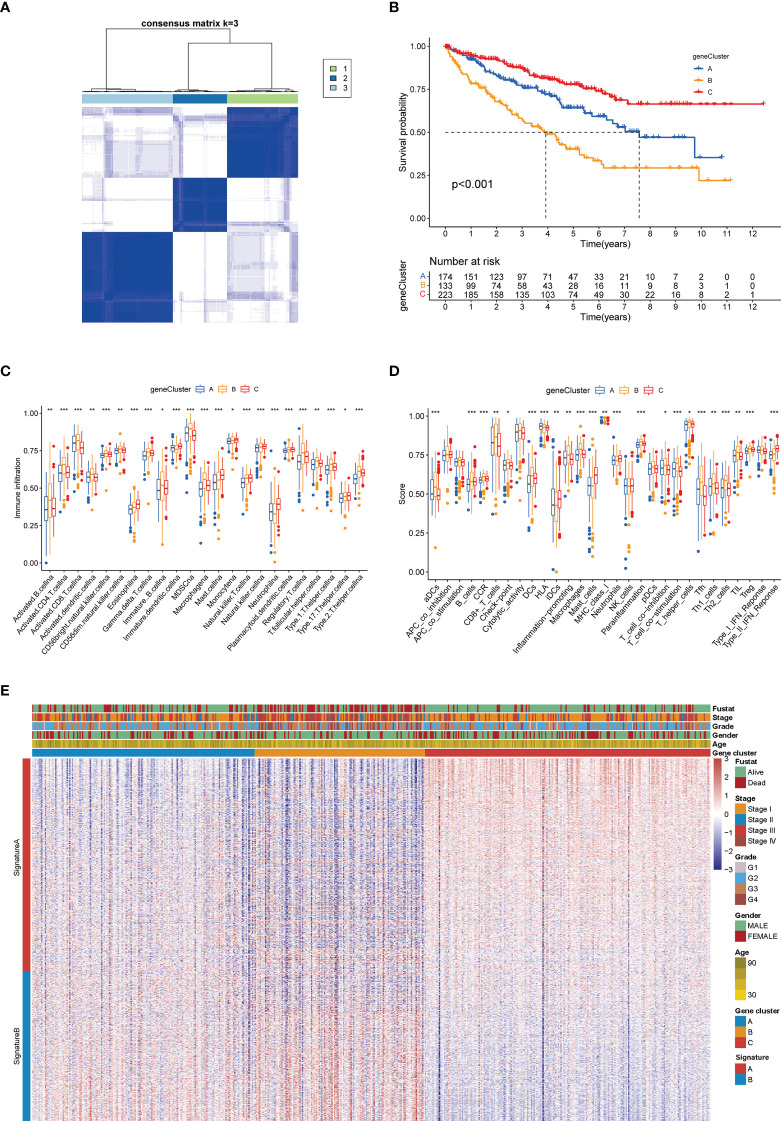
Gene clustering-based grouping of patients with distinct clinical and immune features. **(A)** Heatmap of the consensus matrix depicting the proper clusters (k = 3) and correlation area. **(B)** Kaplan–Meier survival curves of three subgroups. Analysis of immune cell infiltration level **(C)** and immune-related function **(D)** between three subgroups. **(E)** Heatmap of different clinicopathological features in three subgroups (*p < 0.05; **p < 0.01; ***p < 0.001).

Like the CAG-based grouping of ccRCC patients, we subdivided the patients into three groups (designated as A, B, C; N_A_ = 174, N_B_ = 133, N_C_ = 223) using consensus clustering analysis, based on the gene clustering of DEGs achieved above **(** **Figure 6A** **)**. The cumulative distribution function (CDF) curves **(** **Figure S3A** **)** and K-means ranging from 2 to 4 **(** **Figure S3B** **)** showed that the proper gene clustering number was 3. The following survival analysis provided a distinctly separated surviving pattern among three subgroups of patients **(** **Figure 6B** **)**. Similarly, the tumor immune microenvironment (TIM) characteristics of each subgroup were demonstrated in the form of immune cell infiltration **(** **Figure 6C** **)** and immune-related function analysis **(** **Figure 6D** **)**. As the results indicated, nearly all types of immune cells and all processes of immune-related function turned out to be significantly differentially regulated in three subgroups (*p* < 0.05). In the meantime, the clinicopathological feature distinctions between subgroups were also explored and are shown in the heatmap **(** **Figure 6E** **)**. Besides, we made a reverse verification by detecting the CAG level in three subgroups, and the results showed that all CAGs were remarkably dysregulated among subgroups (*p* < 0.01) **(** **Figure S3C** **)**, which further validated the dominant influence of CAG patterns on the DGE grouping of patients (*p* < 0.05 was considered statistically significant).

### Establishment and validation of the cuproptosis score in the KIRC cohort

**Figure 7:**
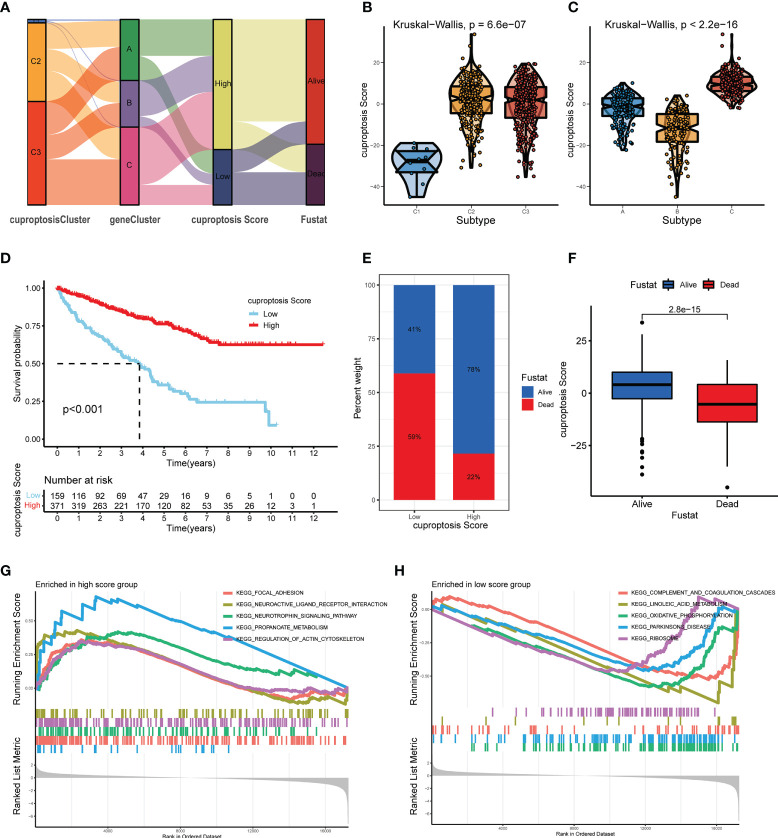
Construction and verification of the cuproptosis score for ccRCC patients from TCGA database. **(A)** Distribution of ccRCC patients in groups based on CAGs, DEGs, cuproptosis score, and surviving status. **(B, C)** Cuproptosis score of diverse subgroups based on CAGs **(B)** and DEGs **(C)**. **(D)** Kaplan–Meier survival curves of ccRCC patients with low and high cuproptosis scores. **(E)** Proportion of dead or alive patients in low and high cuproptosis score groups. **(F)** Overall level of cuproptosis score for patients in alive and dead groups. **(G, H)** KEGG pathway analysis on patients in low and high cuproptosis score groups (*p* < 0.05 *; *p* < 0.01 **; *p* < 0.001 ***). CAGs, cuproptosis-associated genes; DGEs, differentially expressed genes.

Combining the CAG-grouping and DEG-grouping systems, we developed a novel scoring system, called cuproptosis score, as a method to evaluate the prognosis of ccRCC patients. The distribution of ccRCC patients in the CAG-grouping, DEG-grouping, and cuproptosis scoring systems is displayed in **Figure 7A**, as well as the surviving status. Accordingly, the reverse validation was performed, and the violin plots showed that cuproptosis rightly distinguished CAG subgroups and DEG subgroups (*p* < 0.001) **(** **Figures 7B, C** **)**. Then, relying on the calculated cuproptosis score, each patient in the validation cohort was analyzed for survival probability and the results demonstrated that a higher cuproptosis score earned patients significantly more surviving expectation (cutoff value = -4.379531, **Figure 7D** **)**. Meanwhile, the proportion of surviving status (dead or alive) of patients in the high-score and low-score groups is recorded in **Figure 7E**. The overall cuproptosis scores of dead or alive patients are also shown in **Figure 7F**. Moreover, we sought to reveal the potential vital biological processes associated with the cuproptosis scoring system. The results of the KEGG pathway analysis from GSEA showed that in the high-score and low-score groups, two different sets of top five pathways were enriched, respectively **(** **Figures 7G, H** **)**. Therefore, the subsequent validation and exploration of the cuproptosis scoring system demonstrated the prominent significance of cuproptosis in ccRCC.

In the meantime, to compare the characteristics of cuproptosis in non-clear cell RCCs, we conducted parallel analyses on 285 cases of papillary RCC (pRCC) patients from TCGA database. Patients were divided into diverse subgroups based on their cuproptosis-related genetic features. Similarly, patients from different subgroups were subjected to the differential analysis and survival estimation, and the generated cuproptosis scores were applied in the survival analysis as well. However, it turned out that the CAG-based grouping system failed to distinguish high- and low-risk pRCC patients, neither did the cuproptosis score show satisfying predictive capability. The results are shown in **Figure S4** (*p* < 0.05 was considered statistically significant).

### Prognostic value and genetic mutation correlation of the cuproptosis score in ccRCC patients

**Figure 8:**
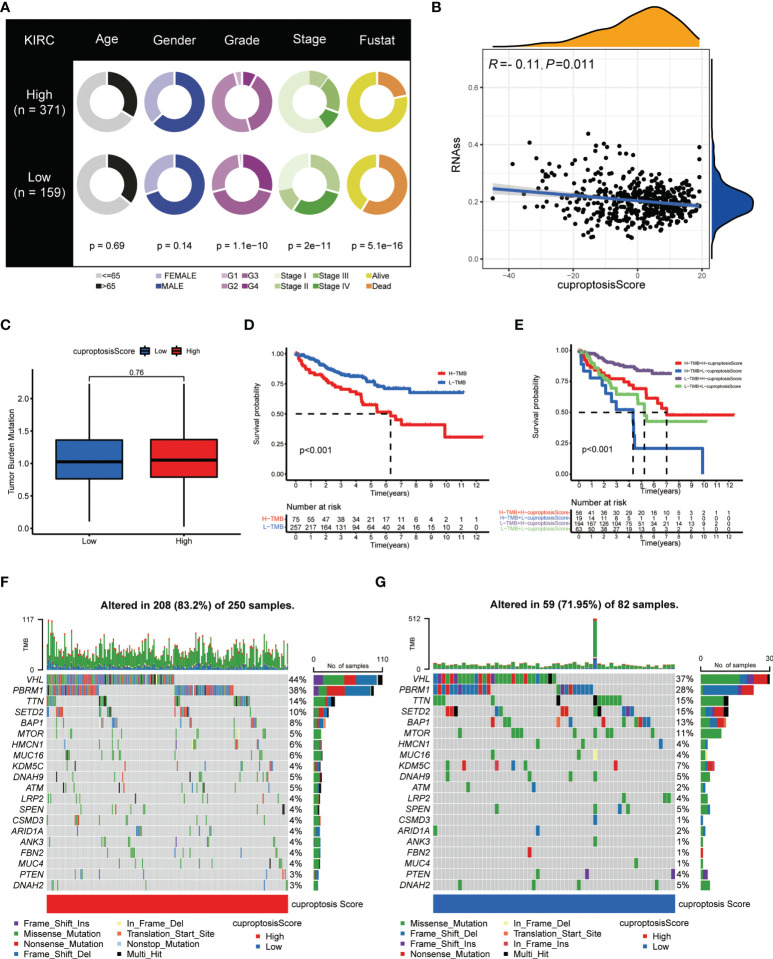
Clinical and genetic correlation of cuproptosis score. **(A)** Clinicopathological information of ccRCC patients in low and high cuproptosis score groups. **(B)** Spearman’s correlation analysis on cuproptosis score and stem cell level. **(C)** Relationships between cuproptosis score and TMB. **(D)** Kaplan–Meier survival curves of patients with high and low TMB. **(E)** Kaplan–Meier survival curves of patients with diverse TMB and cuproptosis scores. **(F, G)** Genetic mutation landscape of patients with high **(F)** and low **(G)** cuproptosis scores. TMB, tumor mutation burden.

Upon the construction and validation of the cuproptosis score, we immediately devoted the scoring system into usage on ccRCC patients from TCGA-KIRC database. First, complete clinicopathological features, including age, gender, tumor grade, cancer stage, and surviving status (Fustat) of patients with high and low cuproptosis scores, are as shown in **Figure 8A**. Detailed clinical and tumoral information (e.g., TNM stage) and their association with cuproptosis are shown in **Table 1**. The cuproptosis score was then investigated on its relevance with cancer stem cell (CSC) score, and Spearman’s correlation analysis observed a negatively correlated relationship between cuproptosis score with the mRNA level of stem cells (r = – 0.11, *p* < 0.05) **(** **Figure 8B** **)**. Since tumor mutation burden (TMB) was confirmed to be tightly associated with the immunotherapy and cancer progression, we explored the prognostic significance of TMB in ccRCC. After granting each patient with a certain TMB score, we divided patients into high- and low-score groups based on the median TMB score **(** **Figure 8C** **)**. The survival analysis (cutoff value = -4.379531) showed that patients with a higher TMB score had a prominently lower OS probability **(** **Figure 8D** **)**, which made it an independent risk factor for ccRCC patients. In the meantime, survival analysis on patients’ PFS obtained similar results (**Figure S2C**). Next, a combination of TMB score and cuproptosis score also turned out be an efficient prognostic factor for the outcomes of patients. As shown in **Figure 8E**, the pattern of two scoring systems in patients effectively distinguished patients in terms of long-term survival. Patients concurrently with a low TMB score and a high cuproptosis score tended to obtain an optimal surviving time and status (the purple curve). Additionally, we explored the somatic mutation landscape of patients when grouping them with cuproptosis score. The heatmaps presented dramatically different mutation incidences in the high-score group (83.2%) and low-score group (71.95%) **(** **Figures 8F, G** **)**. Especially, the mutation incidence and type were quite different in the most common mutative genes VHL (44% vs. 37%) and PBRM1 (38% vs. 28%) in ccRCC, with a nearly average distribution in VHL but a dominant proportion of missense mutation in PBRM1. These results demonstrated that cuproptosis score was intimately correlated with tumor mutation situation and held an equivalent value in predicting the prognosis of ccRCC patients to existing methods (*p* < 0.05 was considered statistically significant).

**Table 1:** Relationship between cuproptosis score and clinicopathological features of KIRC in TCGA.

Covariates	Total	High	Low	P value
		No. (%)	No. (%)	
**Age (years)**				0.6842
**≤**65	348 (65.66%)	246 (66.31%)	102 (64.15%)	
**>**65	182 (34.34%)	125 (33.69%)	57 (35.85%)	
**Gender**				0.1264
Female	186 (35.09%)	138 (37.2%)	48 (30.19%)	
Male	344 (64.91%)	233 (62.8%)	111 (69.81%)	
**Status**				5.00E-04
Alive	357 (67.36%)	291 (78.44%)	66 (41.51%)	
Dead	173 (32.64%)	80 (21.56%)	93 (58.49%)	
**Grade**				5.00E-04
G1	14 (2.64%)	14 (3.77%)	0 (0%)	
G2	227 (42.83%)	186 (50.13%)	41 (25.79%)	
G3	206 (38.87%)	140 (37.74%)	66 (41.51%)	
G4	75 (14.15%)	29 (7.82%)	46 (28.93%)	
Unknown	8 (1.51%)	2 (0.54%)	6 (3.77%)	
**T classification**				5.00E-04
T1	271 (51.13%)	223 (60.11%)	48 (30.19%)	
T2	69 (13.02%)	42 (11.32%)	27 (16.98%)	
T3	179 (33.77%)	104 (28.03%)	75 (47.17%)	
T4	11 (2.08%)	2 (0.54%)	9 (5.66%)	
**N classification**				0.003
N0	239 (45.09%)	166 (44.74%)	73 (45.91%)	
N1	16 (3.02%)	5 (1.35%)	11 (6.92%)	
Unknown	275 (51.89%)	200 (53.91%)	75 (47.17%)	
**M classification**				5.00E-04
M0	420 (79.25%)	316 (85.18%)	104 (65.41%)	
M1	78 (14.72%)	35 (9.43%)	43 (27.04%)	
Unknown	32 (6.04%)	20 (5.39%)	12 (7.55%)	
**TNM stage**				5.00E-04
I	265 (50%)	220 (59.3%)	45 (28.3%)	
II	57 (10.75%)	37 (9.97%)	20 (12.58%)	
III	123 (23.21%)	77 (20.75%)	46 (28.93%)	
IV	82 (15.47%)	36 (9.7%)	46 (28.93%)	
Unknown	3 (0.57%)	1 (0.27%)	2 (1.26%)	

### Indicative role of the cuproptosis score in the TME and immunotherapy of ccRCC

**Figure 9:**
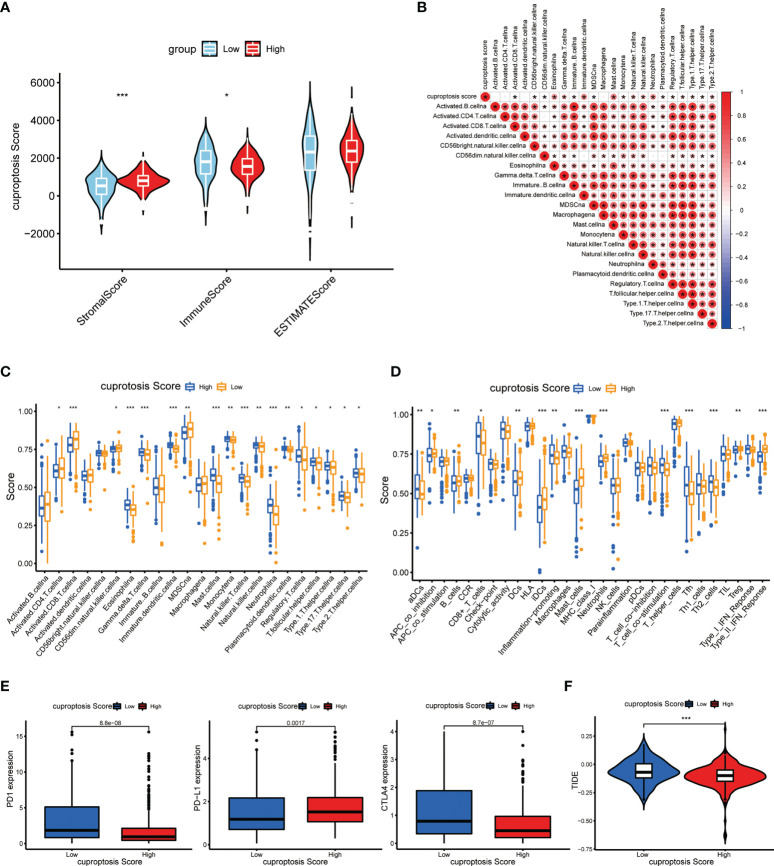
Immune and immunotherapeutic correlation of cuproptosis score. **(A)** Tumor purity of patients with high and low cuproptosis score. **(B)** Correlations between immune-related cells and cuproptosis score. **(C, D)** Abundance of immune cell infiltration **(C)** and immune-related functions **(D)** in patients with high and low cuproptosis scores. **(E)** Abundance of immunotherapy markers in patients with high and low cuproptosis scores. **(F)** TIDE in different cuproptosis score groups (*p* < 0.05 *; *p* < 0.01 **; *p* < 0.001 ***. NS, not significant).

The status of the TME was another imperative factor considered to affect the immune cell infiltration level and immunotherapy treating response of cancer. Therefore, we applied the ESTIMATE algorithm (10) to evaluate the association of cuproptosis score with the TME from three perspectives, namely, stromal score, immune score, and ESTIMATE score. As shown in **Figure 9A**, the stomal score presented significantly higher in the high cuproptosis score group (*p* < 0.001) while the immune score was quite lower (*p* < 0.05). To explore the differences in stromal cells and immune cell signature genes between the two groups, we performed a statistical analysis of differentially expressed genes between the high-score group and the low-score group **(** **Table S1** **)**. Moreover, the genetic differences in stromal signature and immune signature were sorted out **(** **Table S2** **)**. As mentioned before, the cuproptosis score was analyzed to be negatively correlated with the CSC mRNA level, and the high-score patients were also characterized with more frequent genetic mutations in VHL and PBRM1 genes. The CSC abundance normally determined the stromal composition and level of stromal cells in the TME, which together with the diverse gene mutation frequencies explained the significantly elevated stromal score of the high cuproptosis score group. Besides, the correlation analysis revealed a strong association of cuproptosis score with the infiltration level of various immune cells in the TME **(** **Figure 9B** **)**.

As described before, we also investigated the immune infiltration and associated function in high-score and low-score groups. As expected, there was a significantly dysregulated landscape of immune cell infiltration level and immune-related function activity between groups **(** **Figures 9C, D** **)**. Moreover, we made an analysis on essential markers of immunotherapy including cytotoxic T-lymphocyte-associated antigen 4 (CTLA-4) and programmed death 1/programmed death ligand 1(PD-1/PD-L1), which have been proved to be effective immune checkpoint (ICP) inhibitors. It showed that the expression levels of PD-1 and CTLA-4 were dramatically higher in the low cuproptosis score group, while the PD-L1 level presented a reverse discrepant trend **(** **Figure 9E** **)**. On this basis, we suspected that the PD-1 inhibitor is more effective in the low-score group and the PDL-1 inhibitor is more effective in the high-score group. TIDE can be used to evaluate the potential clinical efficacy of immunotherapy in different cuproptosis score groups. The higher the TIDE prediction score, the higher the likelihood of immune evasion, suggesting that patients are less likely to benefit from ICI treatment. As shown in **Figure 9F**, the TIDE score in the high-score group was lower than that in the low-score group, suggesting that patients with high scores may benefit more from ICI treatment than those with low scores. These findings may indicate that the process of cuproptosis connects with certain immune characteristics in the TME of ccRCC, and patients with low cuproptosis displayed relatively more immune abundance (*p* < 0.05 was considered statistically significant).

### Estimation of cuproptosis score-related sensitivity to comprehensive therapies in ccRCC

**Figure 10:**
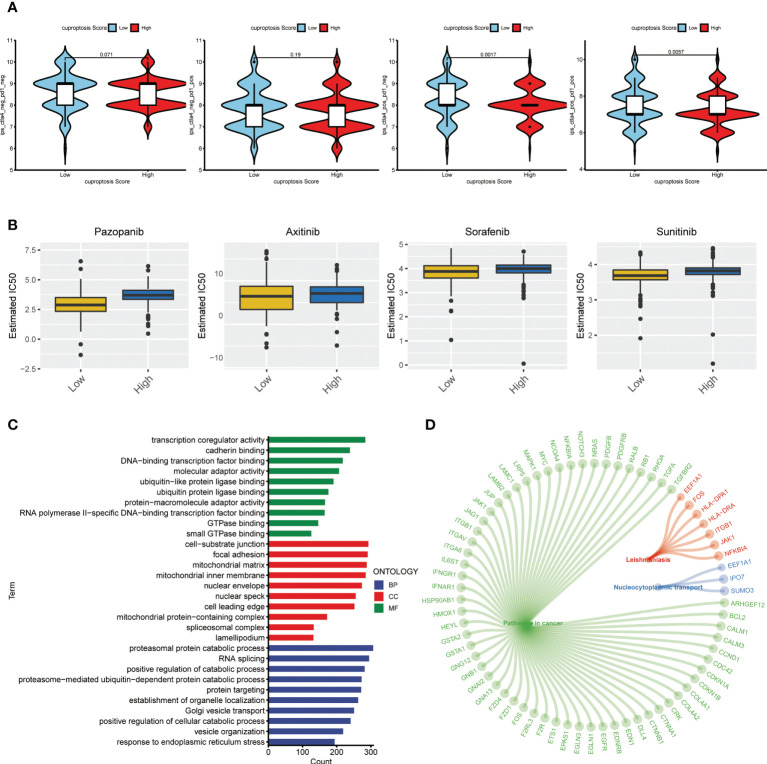
Therapeutic response correlation of the cuproptosis score in ccRCC. **(A)** IPS level in the high and low cuproptosis score groups. pos: positive; neg: negative. **(B)** Estimated treating response of agents in patients with high and low cuproptosis scores. **(C)** GO analysis on DEGs from patients with high and low cuproptosis scores. **(D)** Circle plot of representative pathways and hub genes. IPS: immunophenotype score. GO: gene ontology. DEGs, differentially expressed genes.

The treating strategy for advanced ccRCC patients mainly includes targeted treatment and immunotherapy. The individual difference in treating response is an outstanding concern among the patient population, which enormously affects their long-term survival. Hence, we explored the treating response of ccRCC patients with different cuproptosis scores. First, to evaluate the sensitivity to immunotherapy, each patient from TCGA-KIRC database was given a calculated IPS score to identify the immunotherapy signature level. The results showed that the group of low cuproptosis score had a significantly higher IPS score with a positive CTLA-4 signature compared to the high-score group **(** **Figure 10A** **)**, which indicated that patients with a lower cuproptosis score could be more sensitive to the ICP treating strategy. Next, we made an estimation on IC50s of common small molecular targeted drugs and depicted the landscape of drug sensitivity in ccRCC **(** **Figure 10B** **)**. As a result, we found that patients from the high score group exhibited an active response to the small molecular targeted drugs, which were also known as the tyrosine kinase inhibitors (TKIs) such as sunitinib or pazopanib. Besides, after obtaining the DEGs between groups of low and high cuproptosis scores as previously described, we conducted a similar functional enrichment analysis on them. The result of the GO analysis showed that these DEGs were tightly associated with transcriptional regulation and protein binding in terms of molecular function (MF) and catabolic processes in terms of biological process (BP) **(** **Figure 10C** **)**. The KEGG analysis then revealed the most relevant potential pathways, among which pathways in cancer accounted for the dominant proportion. The vital genes involved in the associated pathways were also identified and displayed in the circle plot **(** **Figure 10D** **)** (*p* < 0.05 was considered statistically significant).

### Prominent anticancer role of FDX1 in ccRCC

**Figure 11:**
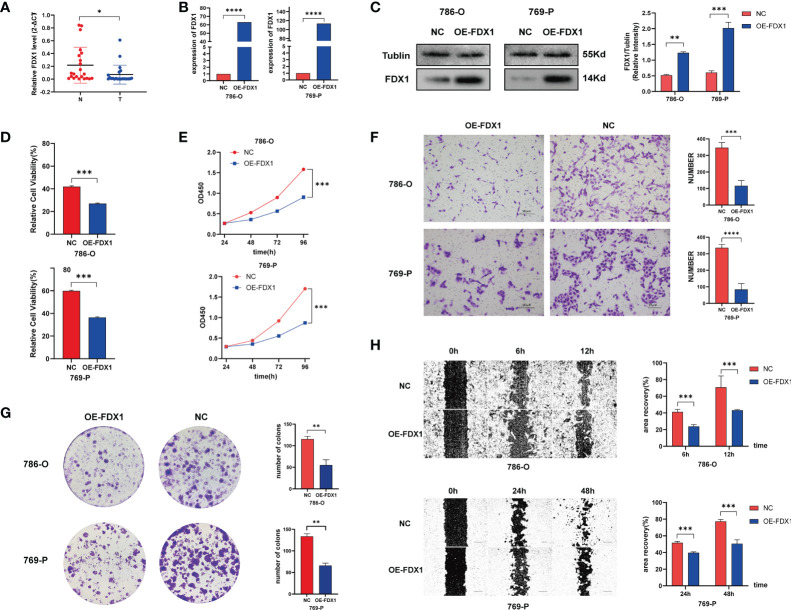
FDX1 regulates cuproptosis to suppress the progress of ccRCC. **(A)** FDX1 mRNA level in 23 paired clinical ccRCC samples. Overexpressing efficiency of FDX1 in ccRCC cell lines validated by q-PCR **(B)** and Western blot **(C)**. **(D)** Relative viability of cells after being treated with elesclomol (20 nM) for 12 h. **(E–H)** CCK 8 assay **(E)**, colony formation assay **(F)**, Transwell assay **(G)**, and wounding healing assay **(H)** on ccRCC cells after transfection of plasmid vector carrying FDX1 (*p* < 0.01 **; *p* < 0.001 ***; *p* < 0.0001 ****).

To further explore the function of cuproptosis in ccRCC, we conducted an experimental investigation using ccRCC clinical samples and cell lines, choosing FDX1 as the hub CAG as indicated in the study of Tsvetkov et al. First, the results of q-PCR on 22 paired clinical ccRCC samples revealed a prominently higher expression of FDX1 in the normal tissues tumors compared to *p* < 0.05 **(** **Figure 11A** **)**, consistent with a previous finding from an online analysis. Next, to make a deeper understanding on the role of FDX1 in the development of ccRCC, we successfully constructed FDX1-overexpression cell lines *via* transfection of plasmid vectors, which was validated by results of qRT-PCR **(** **Figure 11B** **)** and Western blot **(** **Figure 11C** **)**. Then, cells were treated with elesclomol (20 nM) for 12 h to observe the killing effects of copper ionophore-induced cell death. As a result, the copper overloading exerted more lethal influences on cells from OE-FDX1 groups (**Figure 11D**). It suggested that an upregulated expression of FDX1 made cells more vulnerable to intracellular copper accumulation. Next, the result of CCK8 revealed that FDX1 overexpression significantly repressed the growth of both 786-O and 769-P cells (*p* < 0.001, **Figure 11E**). Similarly, the colony formation assay illustrated that higher FDX1 levels alleviated the proliferative trending of cells (*p* < 0.01, **Figure 11F**). In the meantime, we asked if FDX1 affected the aggressive properties of ccRCC cells. As indicated in **Figure 11G**, the Transwell assay showed that both 786-O-OE and 769-O-OE cells displayed significantly decreased migration capability, compared to the control groups (*p* < 0.001). Finally, the wounding healing assay achieved consistent results **(** **Figure 11H** **)**, which depicted the suppressive effect of FDX1 on the invasiveness of tumor cells. Taken together, these results may suggest that FDX1, a hub cuproptosis-related gene, may play a vital anticancer role in the progress of ccRCC (*p* < 0.05 was considered statistically significant).

## Discussion

Generally, copper ion is an important cofactor for enzymes that function in energy generation, cellular metabolism, iron acquisition, oxygen transportation, and numerous biological processes (11). Free intracellular copper is commonly restricted to an extraordinarily low level by the cellular homeostasis system, which attracts great attention on the regulation of transporting machinery and disposition of copper (12). For a long time, copper ionophores were applied as small copper-binding molecules to transport copper into the cell. It was then found that in the copper ionophore–resulted cell death, the accumulation of intracellular copper was responsible for the cellular toxicity, instead of the chaperones themselves (13), and this form of cell death was recognized as copper-induced cell toxicity and death.

As well-researched biological processes, many RCDs have matured academic interpretations and a solid experimental foundation. For example, in the process of ferroptotic cell death, the metal ion Fe serves as the hub initiator, overloaded lipid peroxidation is the vital molecular process, key enzymes mediating the metabolism and the products are indispensable effectors, and the whole oxidative system creates the favorable environment for the series of peroxidative events (14). Yet, we still lack unified cognition of the detailed mechanisms underlying the copper-related toxicity in organisms. Previous studies have offered diverse views about the introduction of apoptosis (15–17), caspase-independent cell death (18, 19), oxidative stress induction (20, 21), and extracellular ATP release (22). In the study of Tsvetkov et al., the researchers held the proposition that cells undergoing aerobic respiration (noted as TCA-cycle active cells) presented an increased level of lipoylated TCA enzymes, which was able to directly bind copper and resulted in the lipoylation of proteins and the loss of Fe-S cluster–containing proteins, finally leading to acute proteotoxic stress and cell death. It has been the first study to build up the exact notion that copper-induced cell death was executed through the function of mitochondria. It also offered detailed insights into the process and consequence of copper-induced cell death, which deserves more studies in the future (6).

Inspired by the study, we performed the present research and aimed to discuss the role of cuproptosis in ccRCC. DLAT, one of the CAGs highlighted in the study, is responsible for encoding the enzyme dihydrolipoamide S-acetyltransferase (DLAT) to serve as an essential component (E2) of the pyruvate dehydrogenase (PDH) complex, which is the protein target of lipoylation. Tsvetkov and colleagues found that the copper directly bound and induced the oligomerization of lipoylated DLAT, which was considered as a direct mechanistic link in the copper-induced cell death. According to the latest report by Tsvetkov and his colleagues, CDKN2A was a key gene that inhibits cuproptosis, which is consistent with our conclusion. Loss-of-function CDKN2A alteration has been identified as a new biomarker for poor patient outcome in multiple cancers. Studies have shown that loss of CDKN2A by deletion of chromosome band 9p21.3 or promoter hypermethylation was frequent in RCC and correlated with poorer survival in all RCC histological subtypes (23). The fundamental mechanism might be that loss-of-function mutations of CDKN2A lead to loss of both proteins encoded by it, p14ARF and p16INK4a, releasing G1/S and G2/M cell-cycle checkpoints and resulting in uninhibited cell proliferation and tumor formation (24). However, CDKN2A alterations were recently reported to be associated with reduced benefit from ICI therapy in urothelial carcinoma, but not with ICI outcomes for RCC (24). The acid dehydrogenase complex (PDH complex) is an important catalytic enzyme in the TCA cycle and plays an important role in the cuproptosis mechanism. However, the direct correlation between the CDKN2A and FHD complex is not clear and has not been clarified in the latest report by Tsvetkov et al. In conclusion, the role and mechanism of CDKN2A alteration in ccRCC should be further explored, which is a long-term investment process.

Next, to characterize ccRCC patients with specific cuproptotic features, we mainly applied the unsupervised/consensus clustering analysis on TCGA-KIRC cohort. In the division of patients based on CAG clustering, DLAT turned out to be of great weight to determine the clusters of patients. Similarly, FDX1, the upstream regulator of protein lipoylation, was also considered the key regulator of copper-induced cell death. In the study of Tsvetkov et al., FDX1 was confirmed to indispensably promote copper-induced cell death as a direct target of elesclomol. In the experimental validation of our study, we used elesclomol as the copper ionophore to manage copper overloading and found that cells in OE-FDX1 groups showed prominently higher killing effects by the drug. The result mechanistically indicated that in cuproptosis, FDX1 was an efficient target for elesclomol to exert copper ionophore-related cell death. Besides, we also found that FDX1 showed a dysregulated expressing pattern in clinical samples, and it was an independent tumor suppressor through relevant functional assays. These findings emphasized again the indispensable role of DLAT and FDX1 in connecting cuproptosis and ccRCC. Nevertheless, to explore the underlying mechanisms of FDX1 regulating cuproptosis in ccRCC, more experiments on the intracellular copper level, mitochondrial ROS induction, and vital protein alterations should be done as further validation (25, 26).

In our research, multiple survival analyses based on the individual CAG, the CAG-derived prognostic model, and the constructed cuproptosis score all showed outstanding capability of distinguishing high- and low-risk ccRCC patients. Apparently, these findings granted cuproptosis tremendous predictive value in the long-term survival of ccRCC patients. Nevertheless, when comparing the cuproptosis featuring of pRCC with ccRCC patients, we found that neither the CAG-based grouping system nor the cuproptosis scoring system exhibited expectant predicting value. In survival analysis, there was no significant difference observed between different clusters of patients. The cuproptosis score did not distinguish pRCC patients with high or low surviving risks as well. Despite the enough samples of pRCC cases from TCGA database, we proposed that cuproptosis may not play a role in non-ccRCC as vital as in ccRCC.

In addition to the survival and clinical correlation, the immune-related characteristics have been thought to be of extraordinary meaning in cancer progression and treatment (27). Following the routine, we made parallel analyses on immune cell infiltration and immune-related functions in CAG-based subgroups, DEG-based subgroups, and cuproptosis score-based subgroups of ccRCC patients, successively. The overall results in subgroups indicated significant associations between TME, immune features, and important CAGs. In CAG-based grouping patients, those from C2 and C3 subgroups seemed to have a lower immune cell infiltration level and less active immune processes, while they behaved better in the long-term survival analysis. Similarly, in the DEG-based subgroups, patients from cluster B showed significantly elevated immune features while they had worse survival outcomes compared to others. This indicated an attractive relationship between TME characteristics of patients and the way we divided them based on cuproptosis-associated genes. Notably, both immune cell infiltration and immune functions displayed distinct levels among diverse subgroups based on CAG and DEG clustering. However, in terms of cuproptosis scoring system, patients with a certain score seemed to obtain inconsistent distribution among all types of immune cells and immune functions. The result may suggest the obscure function of cuproptosis score in immune characteristics, and more specific investigation is needed to explain the meaning of cuproptosis in the tumor microenvironment. Interestingly, previous studies widely reported that copper could exert remarkable impacts on immune response and microenvironment in cancers. Zheng et al. found that when using the disulfiram/copper codelivery system to treat glioblastoma, the efficacy was reinforced *via* its remodeling effect on the immune microenvironment of the glioma (28). Liu and colleagues reported that tetrathiomolybdate (TM), a promising anticancer compound, influenced collagen remodeling and enhanced immune response by depleting intracellular copper in breast cancer (29). Yet, rare studies introduced the immune correlation in copper-induced cell death. It was only reported by Mitra and colleagues that overexposure of copper yielded potential adverse immunotoxicity on health *via* cell cycle rest and apoptosis *in vivo* (30, 31). Therefore, we proposed that the copper-induced cell death/cuproptosis deserved more investigation to figure out its complicated role in TIME.

On the other hand, immunotherapy has occupied an imperative position in cancer treatment, and ccRCC is with no exception (32). Combined with TKI as the first-line treatment option, the representative immunotherapeutic agents constitute the ICP therapy, including nivolumab (PD-1 inhibitor), ipilimumab (CTLA-4 inhibitor), and atezolizumab (PD-L1 inhibitor) (33). Our study found that patients with a low cuproptosis score had significantly higher PD-1 and CTLA4 expression but a lower PD-L1 level. Besides, by calculating IPS for each patient, we found that patients with a low cuproptosis score had a higher IPS score, which indicated that these patients may be more sensitive to the ICP treating strategy. It was not the first time that copper was reported to be correlated with immunotherapy efficacy in cancers. Voli et al. found that copper could upregulate the PD-L1 expression and contribute to cancer immune evasion, while copper chelators were observed to reverse the effect by promoting ubiquitin-mediated degradation of PD-L1 (34). Zhou et al. also reported that a combination of disulfiram with copper stabilized the PD-L1 expression, which induced immunosuppression in the treatment of hepatocellular carcinoma (35). Furthermore, considering the irreplaceable role of targeted therapy in ccRCC treatment (36), we evaluated whether the TKI sensitivity of ccRCC patients was affected by the cuproptosis score, and a surprising difference was observed in the estimated IC50 of sunitinib between high and low score groups. Taken together, the cuproptosis score constructed by us was a potential indication of immunotherapy response and targeted treatment sensitivity in ccRCC patients.

Throughout the study, by applying the ccRCC cohort from TCGA database, we successively divided these patients into several subtypes with a distinct clinical and genetic background based on cuproptosis-related patterns. Directed at the subgroups, we performed multiple analyses and they revealed critical clinical significance and a valuable prognostic role of cuproptosis in ccRCC. To validate these findings, we performed *in vitro* verification using clinical samples and cell lines. Most importantly, we explored the relationship between vital gene FDX1 and copper ionophore-induced cell death in ccRCC, which brought benefits to further understand the cuproptosis in ccRCC. However, we did not dig into the underlying mechanisms of other vital CAGs working to influence the clinical and molecular characteristics of ccRCC. Besides, we failed to make conclusions on the specific association of cuproptosis score and TIME. Hence, we call for a deeper investigation into the copper-induced cell death in future studies, and we believe that the metal ion-associated life events have great potential to be targeted in human cancers.

## Conclusion

Our study has some advantages compared with other published signatures. First of all, most studies of ccRCC based on TCGA and GEO databases focus on constructing risk models using gene sets. There are few studies on cluster analysis and scoring system based on specific gene sets. Secondly, in addition to constructing the traditional risk signature, we also constructed a special cuproptosis scoring system. This novel scoring system can not only accurately predict the prognosis of patients but also assess the sensitivity of individual ccRCC patients to chemotherapy drugs and immunotherapy. This scoring system makes our research more innovative and superior than traditional signature research. In a word, we discovered the intimate relationship of cuproptosis and ccRCC and provided the cuproptosis score for further application in the characterization of clinical background and immune features, and the decision on treatment for ccRCC patients.

## Data availability statement

The original contributions presented in the study are included in the article/**Supplementary Material**. Further inquiries can be directed to the corresponding authors.

## Ethics statement

The studies involving human participants were reviewed and approved by Ethical Committee of the First Affiliated Hospital of Nanjing Medical University. The patients/participants provided their written informed consent to participate in this study.

## Author contributions

BW and QS conceived the project. YW contributed to the data acquisition, analysis, and interpretation, and manuscript writing. BW conducted the experiments and revised the manuscript. All authors read and approved the submitted manuscript. JQ and PS provided the funding and supervised the whole study. All authors contributed to the article and approved the submitted version.

## Funding

This study was supported by the Key Research and Development of Jiangsu Province (Grant No. BE2018749) and the National Natural Science Foundation of China (Grant No. 82002697).

## Conflict of interest

The authors declare that the research was conducted in the absence of any commercial or financial relationships that could be construed as a potential conflict of interest.

## Publisher’s note

All claims expressed in this article are solely those of the authors and do not necessarily represent those of their affiliated organizations, or those of the publisher, the editors and the reviewers. Any product that may be evaluated in this article, or claim that may be made by its manufacturer, is not guaranteed or endorsed by the publisher.

## Supplementary material

Supplementary Figure 1 The entire analytical process of the study.

Supplementary Figure 2
**(A, B)** Results of enrichment analysis including GO and KEGG. **(C)** PFS curves of ccRCC patients with low and high cuproptosis score. PFS, progression-free survival.

Supplementary Figure 3
**(A)** The cumulative distribution function (CDF) curves and **(B)** K-means ranging from 2-4 showed the proper gene clustering number was 3. **(C)** CAGs were remarkably dysregulated among subgroups. (*p* < 0.05 *; *p* < 0.01 **; *p* < 0.001 ***).

Supplementary Figure 4
**(A)** The cumulative distribution function (CDF) curves and K-means curve. **(B)** Heatmaps of consensus matrix depicting the possible clusters (k=2, 3, 4) and correlation areas. **(C)** Survival analyses on pRCC patients based on three ways of clustering. **(D)** Differential analyses on gene sets based on three ways of clustering. **(E)** Survival analysis on pRCC patients based on cuproptosis score. pRCC: papillary renal cell carcinoma.
