# Supplementary figures and images for: Comprehensive investigation into cuproptosis in the characterization of clinical features, molecular characteristics, and immune situations of clear cell renal cell carcinoma
Source: Front Immunol. 2022 Oct 6;13:948042. doi: 10.3389/fimmu.2022.948042 (PMC9582538; doi:10.3389/fimmu.2022.948042)

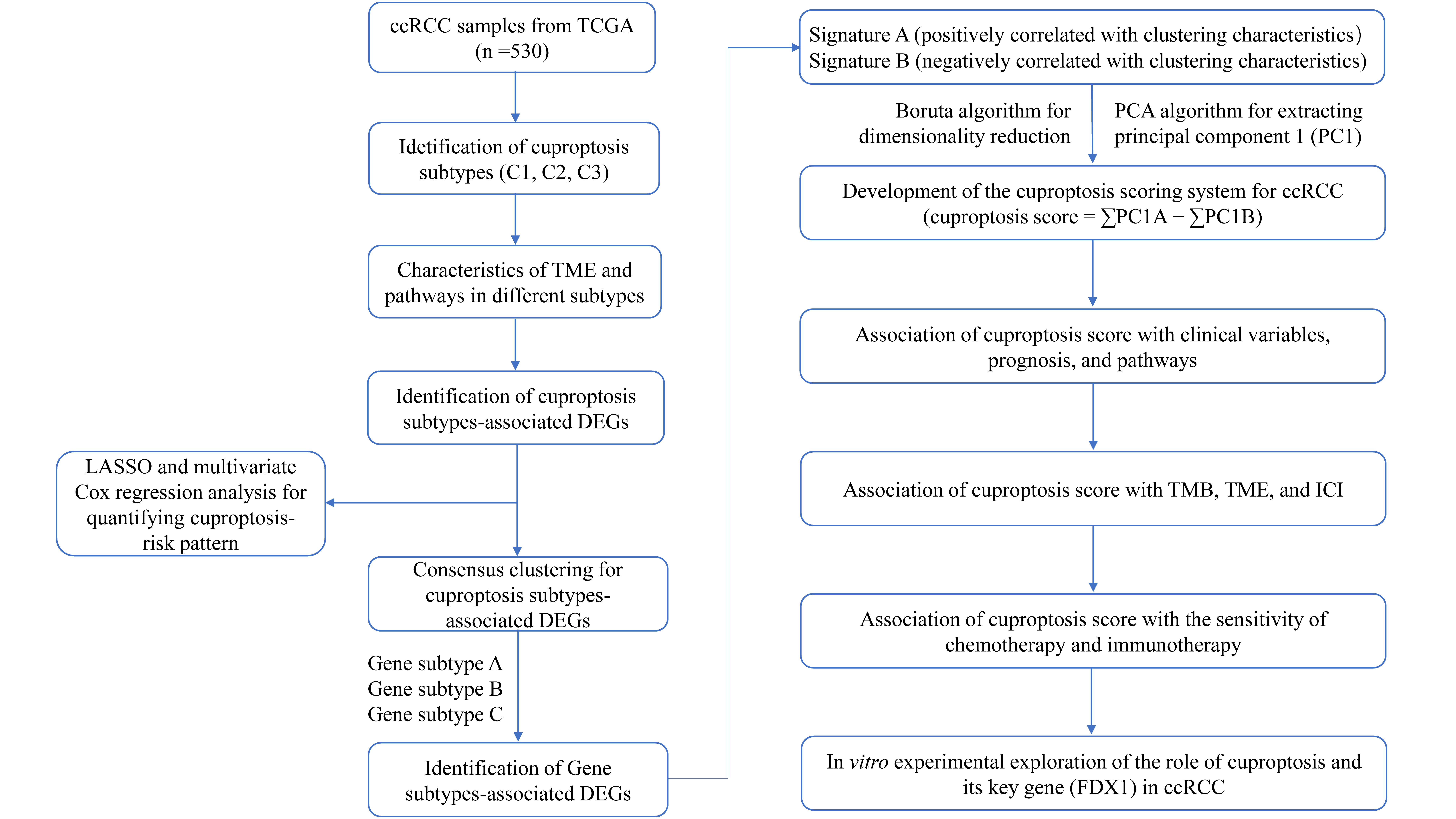

Supplement: Supplementary Figure 1 — The entire analytical process of the study. [file Image_1.tif]

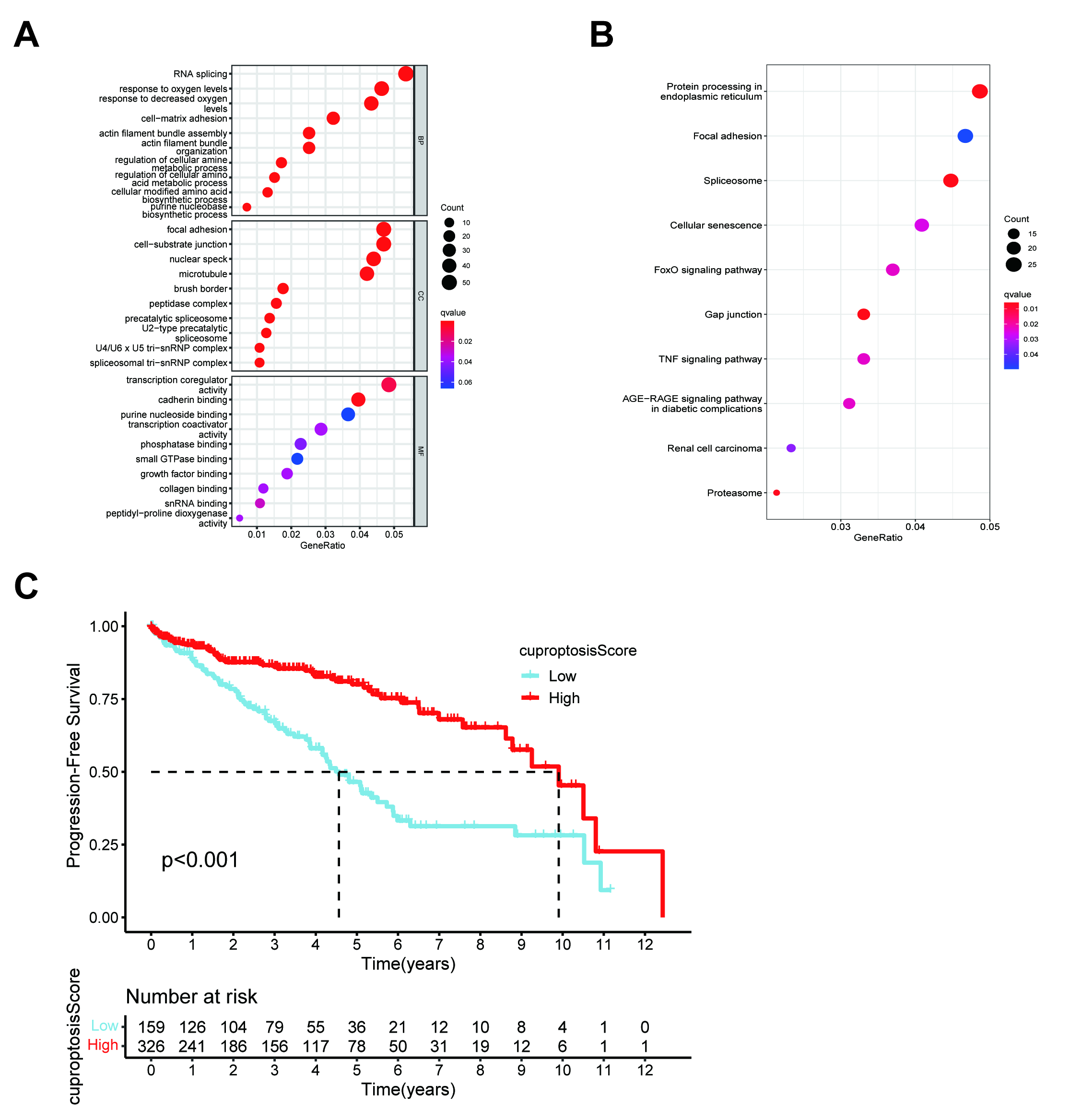

Supplement: Supplementary Figure 2 — (A, B) Results of enrichment analysis including GO and KEGG. (C) PFS curves of ccRCC patients with low and high cuproptosis score. PFS, progression-free survival. [file Image_2.tif]

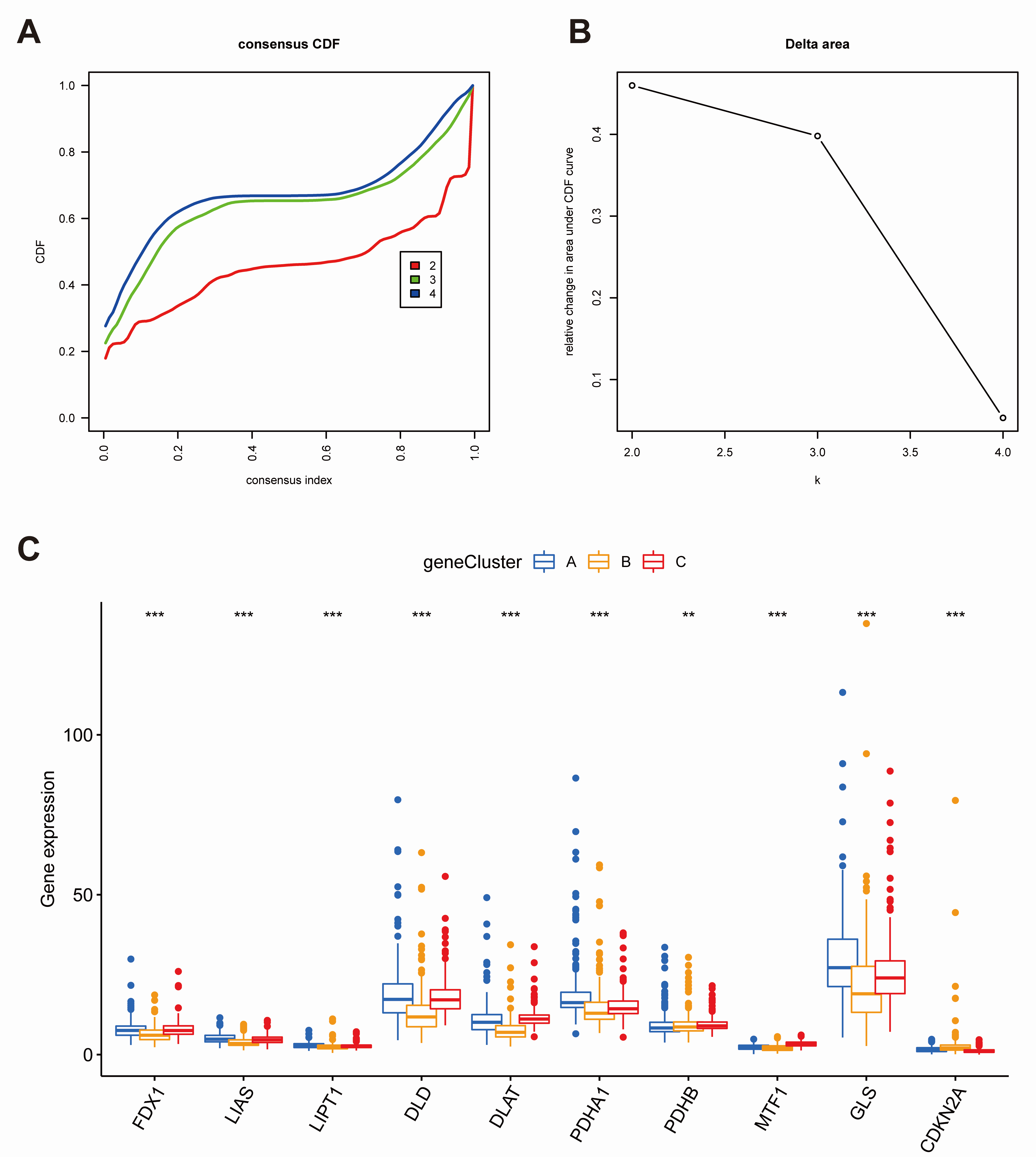

Supplement: Supplementary Figure 3 — (A) The cumulative distribution function (CDF) curves and (B) K-means ranging from 2-4 showed the proper gene clustering number was 3. (C) CAGs were remarkably dysregulated among subgroups. (p < 0.05 *; p < 0.01 **; p < 0.001 ***). [file Image_3.tif]

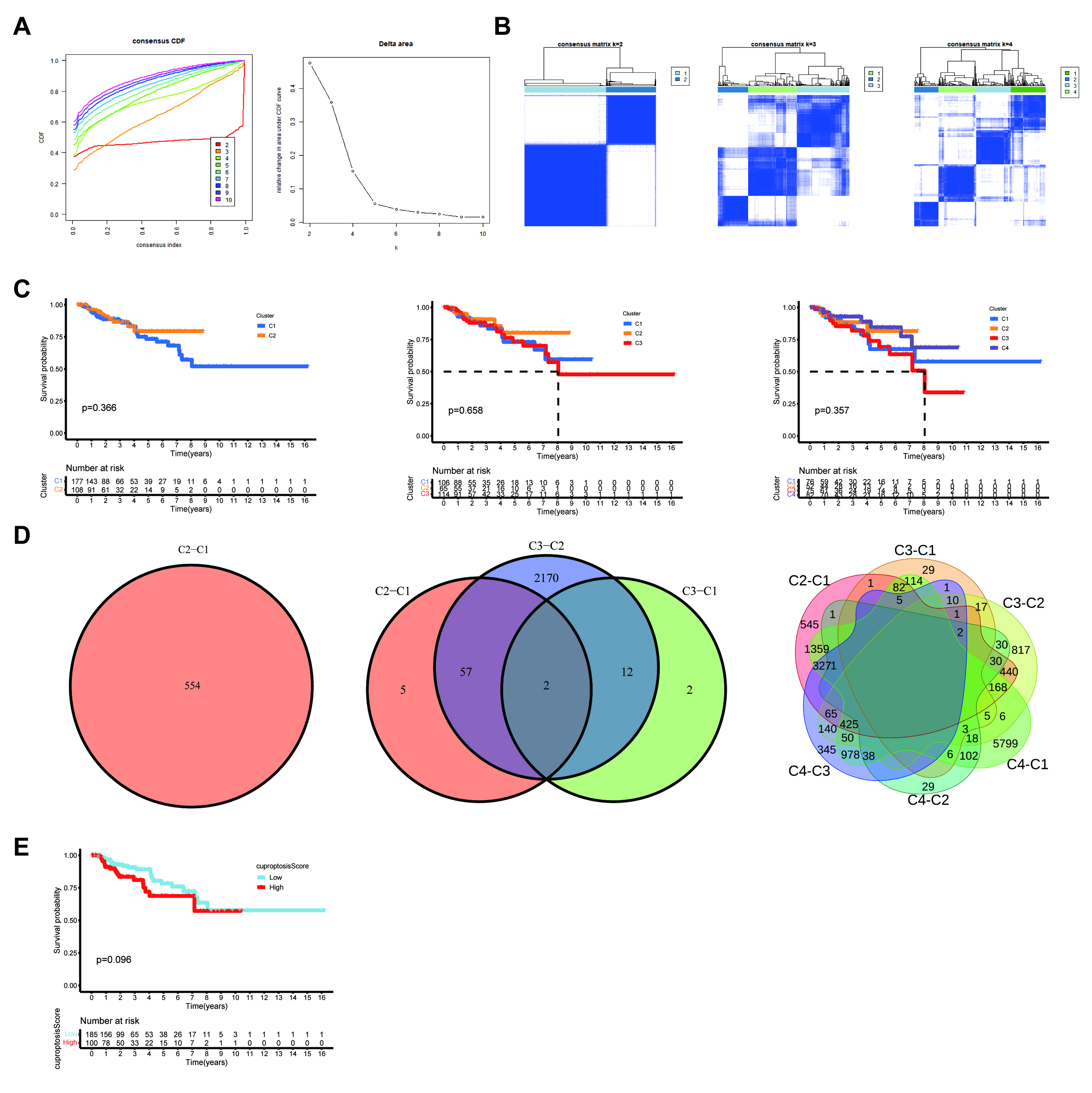

Supplement: Supplementary Figure 4 — (A) The cumulative distribution function (CDF) curves and K-means curve. (B) Heatmaps of consensus matrix depicting the possible clusters (k=2, 3, 4) and correlation areas. (C) Survival analyses on pRCC patients based on three ways of clustering. (D) Differential analyses on gene sets based on three ways of clustering. (E) Survival analysis on pRCC patients based on cuproptosis score. pRCC: papillary renal cell carcinoma. [file Image_4.tif]
